# Adaptation of in vitro cytoadherence assay to *Plasmodium knowlesi* field isolates

**DOI:** 10.1186/1475-2875-9-S2-O13

**Published:** 2010-10-20

**Authors:** Farrah A Fatih, Angela Siner, Atique Ahmed, Lu Chan Woon, Alister Craig, Henry M Staines, Balbir Singh, Sanjeev Krishna, Janet Cox-Singh

**Affiliations:** 1 Centre for infection, St George's University of London, London SW17 0RE, UK; 2 Malaria Research Centre, University Malaysia Sarawak, Kuching, 93150, Malaysia; 3 Pathology Laboratory, Hospital Sarikei, Sarikei, 96100, Malaysia; 4 Molecular Biochemical Parasitology, Liverpool School of Tropical Medicine, L3 5QA, UK

## Background

*P. knowlesi* was the first *Plasmodium* species in which antigenic variation was observed. Variation was due to schizont infected cell agglutination (SICAvar) antigens expressed by the parasite and transported to the exposed surface of the host erythrocyte [1]. PfEMP1 is *P. falciparum's* orthologue of *P. knowlesi's* SICA proteins [2]. In *P. falciparum Pf*EMP1 is associated with infected erythrocytes binding to receptors such as ICAM-1 expressed on the endothelial cells of the host microvasculature. Here, we use a static protein assay [3] to determine if naturally occurring human *P. knowlesi* infections can cause erythrocytes to bind to ICAM-1, VCAM-1 and CD36.

## Materials and methods

Blood samples were collected after obtaining written consent from patients presenting with *P. knowlesi* infection at two well-established study sites in Sarawak, Malaysian Borneo. The samples were washed and cultured *ex vivo* until the majority of parasites had matured to late trophozoite/early schizont stages of development. The parasites were then assayed for their ability to bind potential endothelial ligands. Static assays were carried out with purified proteins, ICAM-1, VCAM-1 and CD36. *P. falciparum* (cloneHB3) was used as a positive control.

## Results

The relative binding of *knowlesi*-patient isolates compared with *P. falciparum* control assays will be presented.

## Conclusion

The results of the binding assays will be presented and discussed in the context of recently described *post-mortem* - findings from a fatal case of *P. knowlesi.*
